# Risk factors and outcome of *Shigella* encephalopathy in Bangladeshi children

**DOI:** 10.1371/journal.pntd.0005561

**Published:** 2017-04-28

**Authors:** Farzana Afroze, Tahmeed Ahmed, Monira Sarmin, Abu SMSB Shahid, K. M. Shahunja, Lubaba Shahrin, Mohammod Jobayer Chisti

**Affiliations:** Nutrition & Clinical Services Division (NCSD), International Centre for Diarrhoeal Disease Research, Bangladesh (icddr,b), Dhaka, Bangladesh; University of California San Diego School of Medicine, UNITED STATES

## Abstract

**Background:**

Although, *Shigella* encephalopathy, a serious extra-intestinal complication of shigellosis, significantly increases the risks of death, data are very limited on predicting factors particularly related to electrolyte profiles in children below five years of age with *Shigella* encephalopathy. Our objective was to determine the clinical as well as laboratory predicting factors and outcome of children with *Shigella* encephalopathy.

**Methodology/Principal findings:**

In this unmatched case-control design, children aged 2–59 months having a positive stool culture for *Shigella* and who had their serum electrolytes been done from July 2012 to June 2015 were studied. Children with *Shigella* encephalopathy, defined as having abnormal mentation, constituted the cases, and those without encephalopathy constituted the controls. During the study period, we identified a total of 541 children less than five years of age, who had *Shigella* in their stool culture. Only 139 children fulfilled the study criteria and among them 69 were cases and 70 were controls. The cases more often had fatal outcome compared to the controls (7% vs. 0%, *P* = 0.02). In logistic regression analysis, the cases were independently associated with shorter duration (1.2 ± 0.4 days) of diarrhea prior to admission, dehydrating diarrhea, sepsis and hyponatremia (p<0.05 for all). Among 139 *Shigella* isolates, *S*. *flexneri* (88/139, 63%) and *S*. *sonnei*(34/139, 24%) were the dominant species. *S*. *dysenteriae* was not isolated throughout the study period. *S*.*sonnei* was more frequently isolated from the cases (24/69, 35%) than the controls (10/70, 14%), whereas the isolation of *S*. *flexneri* was comparable between the groups (40/69, 58% vs 48/70, 69%). A total of 94 (67.6%) isolates were resistant to trimethoprim-sulphamethoxazole, 84 (60.4%) to ciprofloxacin, 66/138 (48%) to ampicillin, 5 (3.5%) to ceftriaxone, 17 (12.2%) to mecillinum and 35 (25%) to azithromycin.

**Conclusions/Significance:**

The case-fatality-rate was significantly higher among the children with *Shigella* encephalopathy compared to those without encephalopathy. Early identification and aggressive management of simple risk factors for *Shigella* encephalopathy may help to reduce morbidity and deaths in such children especially in resource-limited settings.

## Author summary

Bacterial diarrhea due to *Shigella* infection is a common problem in developing countries including Bangladesh. It is one of the major causes of mortality and morbidity particularly in children under five years of age. Encephalopathy, defined by altered mental status, is a life threatening presentation in children having shigellosis. The most common presentations of *Shigella* encephalopathy are seizure, altered consciousness and coma. If the treatment is delayed, deaths in children having *Shigella* encephalopathy are very high. However, we do not have sufficient knowledge about the underlying factors that contribute to encephalopathy, particularly the laboratory characteristics of such children. Thus, we wanted to know the risk factors and outcome of children with *Shigella* encephalopathy. Among a total of 139 Bangladeshi under five children with shigellosis enrolled for 36 months, those who had *Shigella* encephalopathy were independently associated with dehydrating diarrhea, sepsis, and hyponatremia and had higher case-fatality-rate compared to those without *Shigella* encephalopathy.

## Introduction

*Shigella* is a common cause of bacterial diarrhea and is endemic throughout the world [1], especially in developing countries. It is one of the leading causes of mortality and morbidity [2]. *Shigella* predominately affects children younger than 5 years old [3]. In developing countries of Asia, the incidence rate in this age group is around 13/1000 children annually, which is approximately hundred folds higher than in industrialized countries [4]. Still the burden of the disease is underestimated in developing countries and causes a substantial number of in-patient admissions [4,5].

Among several severe extra intestinal manifestations, encephalopathy is one of the most common complications [6,7]. It has been associated with a higher rate of deaths [8–10]. The most common manifestations of *Shigella* encephalopathy are seizure, altered consciousness and coma [11]. Encephalopathy is usually reversible, but sometimes it can be fulminate and fatal [11]. A lethal form of encephalopathy known as Ekiri syndrome (in Japanese ‘epidemic dysentery’) is characterized by rapidly developing seizure and coma with only mild colitis [12,13]. Although, neurological abnormalities and other extra intestinal manifestations are more common in children with *Shigella dysentery* type 1 infection, all four species of *Shigella* (*S*. *dysenteriae*, *S*. *flexneri*, *S*. *boydii*, and *S*. *sonnei*) have the ability to cause this serious neurological complication [5]. Outbreaks due to *Shigella* infection are difficult to control because of their low infective dose, particularly in areas of poor hygienic conditions with limited access to clean and potable water [14,15].

The pathophysiology of *Shigella* encephalopathy has not been elucidated. It is postulated that shiga toxin production is not essential for this lethal complication [16,17]. Shiga toxin, first described in 1903, only produced by *S*. *dysenteriae* type 1, however, S. *flexneri*, and *S*. *sonnei* do not produce shiga toxin as demonstrated by toxin neutralization and DNA hybridization studies [16]. Often encephalopathy results in the early development of brain edema [8,13,17,18]. During the acute stage of encephalopathy serum and cerebrospinal fluid concentrations of inflammatory cytokines such as tumor necrosis factor alpha (TNF-alpha), interleukin-1 (IL-1), and interleukin-6 (IL-6) become elevated [19]. Complementary studies in a murine model also documented similar findings suggesting that host inflammatory response has a major role in the pathogenesis [20,21].

Management of such children is often challenging even in developed countries [22]. Moreover emergence of multidrug resistant strains has made the scenario more complicated [4,23]. Thus early prediction of *shigella* encephalopathy using simple clinical and laboratory characteristics might improve patient outcome, predominantly in developing countries. Even though several clinical characteristics such as lack of breast feeding in neonatal period, dehydrating diarrhea, shorter duration of diarrhea and severe stunting were found to be the risk factors for *Shigella* encephalopathy [11], data are very limited on laboratory predicting factors particularly electrolyte profiles of such children. Therefore, the aim of our study was to determine the clinical as well as laboratory predicting factors and outcome of children with *Shigella* encephalopathy.

## Methodology

### Ethical statement

This study was a retrospective medical record analysis and did not involve any interview with patients or their caregivers. We collected the data anonymously. Importantly, the Institutional Review Board (named as Research Review Committee and Ethical Review Committee) of icddr,b has approved the manuscript.

### Study design

We used an unmatched case-control design for this retrospective analysis. A total of 541 children of either sex, aged 2–59 months, admitted to the study hospital from July 2012 to June 2015 having a positive stool culture for *Shigella* were identified. Among them, 139 children had their serum electrolytes been done at admission and were included in the study. Between them, 69 children with *Shigella* encephalopathy constituted the cases, whereas 70 children with shigellosis having no encephalopathy considered the controls. *Shigella* encephalopathy was defined as any child with shigellosis having disorientation, drowsiness, confusion, convulsion, or coma [11]. We excluded the children who had central nervous system infection based on their cerebro-spinal fluid (CSF) analysis.

Freshly passed fecal samples or rectal swabs of under five children with diarrhea were plated directly onto MacConkey’s agar and *salmonella*-*shigella* (SS) agar, and all *Shigella* strains were isolated and identified following standard microbiological and biochemical methods [24]. Species identification was confirmed by slide agglutination test using commercial *Shigella* antisera kit (Denka Seiken, Tokyo, Japan) [25].

### Study settings

We conducted this study at Dhaka Hospital of icddr,b, the largest diarrheal disease hospital in the world. Every year this hospital provides treatment and care to around 140,000 patients of all age with diarrheal illness with or without associated problems and/or complications. Another description of the hospital has been provided elsewhere [26].

### Patient managements

All children (both cases and controls) received standard management following the hospital guidelines. These include rehydration using oral rehydration solution (ORS) and/or intravenous fluids based on their dehydration status, replacement of ongoing losses, appropriate antimicrobial therapy and other supportive management like proper feeding, micronutrients, vitamins, and minerals when required. Children with encephalopathy were shifted to intensive care unit (ICU) and received supportive care; concurrently we performed all relevant investigations including electrolytes. After initial stabilization and control of seizure, we performed the lumber puncture. In the acute stage, vital signs, input and output, and neurological status were monitored closely. Metabolic status, especially blood glucose and serum electrolyte concentrations were measured serially and corrected when required. Parenteral fluids and pressors were provided to support the systemic circulation and as well as assisted ventilation, as needed.

### Measurements

Case Report Forms (CRFs) were developed and finalized for acquisition of relevant data from the electronic data archive of Dhaka Hospital Patients. Characteristics analyzed included demographic information {age, sex, non-breast fed, poor socioeconomic status, nutritional status [weight for length/height Z scores of WHO median <-3, and or nutritional edema as severe acute malnutrition (SAM), and weight for length/height Z scores of WHO median <- 2 to -3 as moderate acute malnutrition (MAM)], use of antibiotics prior to admission}, clinical features [watery stool (lack of visible blood in stool), diarrhea, vomiting, dehydration, sepsis, pneumonia], admission laboratory characteristics [hypoglycemia (RBS <3.0 mmol/L), hyponatremia (serum sodium < 135.0 mmol/L), hypernatremia (serum sodium > 150.0 mmol/L), hypokalemia (serum potassium < 3.5 mmol/L), hyperkalemia (serum potassium > 5.5 mmol/L), Hypochloremia (serum chloride < 96 mmol/L), hypocalcemia (serum calcium < 2.12 mmol/L), metabolic acidosis (serum TCO_2_ < 17.0 mmol/L), serum creatinine (within normal reference value for age), anemia (hemoglobin <11 gm/dl), features of colitis in stool microscopy (RBC, pus cell >50/HPF, macrophage), stool culture] and outcome.

### Analysis

All data were entered into SPSS for Windows (Version 20.0. Armonk, NY: IBM Corp) and Epi Info (version 7.0, USD, Stone Mountain, GA). The Chi-square test compared differences in proportion. Student’s t-test was used to compare the means of normally distributed data and Mann-Whitney test was used for comparison of data that were not normally distributed. A probability of less than 0.05 was considered statistically significant. Strength of association was determined by calculating odds ratio (OR) and their 95% confidence intervals (CIs). To identify risks for encephalopathy in children with shigellosis, variables were initially analyzed in a uni-variate model, and then risks independently associated with *Shigella* encephalopathy identified using step-wise logistic regression analysis after controlling for the co-variates.

## Results

**Fig 1:**
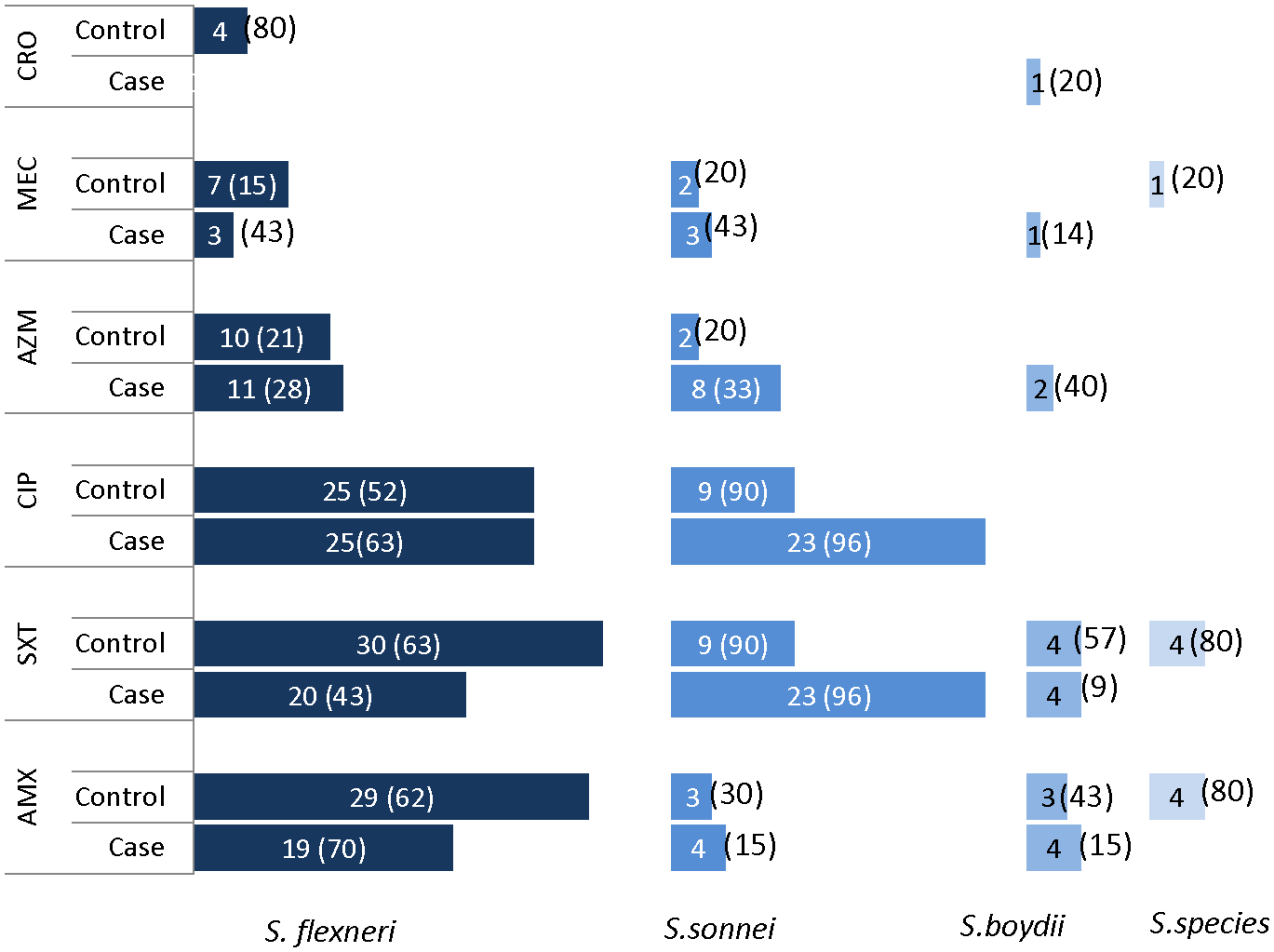
Antimicrobial resistance of *Shigella* isolates among cases and controls.

Of 139 children with shigellosis aged less than five years from July 2012 to June 2015, 69 had *Shigella* encephalopathy and 70 did not have encephalopathy. Children with *Shigella* encephalopathy more often were older and less often had severe acute malnutrition compared to those without encephalopathy (Table 1). They more often presented with dehydrating diarrhea having shorter duration prior to admission, sepsis, hypoglycemia, hyponatremia, and *Shigella sonnei* compared to their counterpart (Tables 1, 2 and 3). A total of five children with *Shigella* encephalopathy died. However, there was no death in controls, and the case–fatality-rate was significantly higher among the *Shigella* children with encephalopathy compared to those without encephalopathy (Table 1). *Shigella* isolates from fatal cases were *Shigella boydii* (3/5, 60%) and *Shigella flexneri* (2/5, 40%) respectively. Other variables shown in Tables 1–3 were comparable among the groups. In ‘Fig 1’, we provided the antimicrobial susceptibility of the isolates in cases and controls.

**Table 1:** Clinical characteristics of diarrheal children under five years of age with *Shigella* encephalopathy compared to those without encephalopathy.

Characteristics	Cases (n = 69) %	Controls (n = 70) %	Unadjusted OR	Unadjusted 95% CI	*p value*
Age (median, IQR)	24 (15, 30)	12 (6.5, 20.5)	-	-	0.00
Male gender	43 (62)	51 (73)	0.6	0.3–1.2	0.25
Non breast-fed	38 (67)	28 (64)	1.1	0.5–2.6	0.91
Poor socio-economic status	24/27 (89)	35/37 (94)	0.4	0.0–2.7	0.39
Watery stool	44/68 (65)	41/67 (61)	1.2	0.6–2.3	0.80
Use of antibiotics prior to admission	22/61 (36)	12/30 (40)	0.8	0.3–2.0	0.89
Duration of diarrhea (mean ± SD)	1.4 ± 0.5	1.9 ± 0.7	-	-	0.00
Duration of vomiting (mean ± SD)	1.2 ± 0.4	1.5 ± 0.6	-	-	0.31
Dehydration (some/severe)	43 (63)	28 (41)	2.4	1.2–4.8	0.01
SAM	13 (19)	30 (43)	0.3	0.1–0.6	0.00
MAM	18 (26)	24 (35)	0.6	0.3–1.3	0.35
Pneumonia	8/65 (12)	11/67 (14)	0.7	0.2–1.9	0.67
Sepsis	29 (43)	9 (13)	4.8	2.0–11.2	0.00
Outcome (death)	5 (7)	0 (0)	-	-	0.02

Figures represent n (%), unless specified. OR: odds ratio. CI: confidence interval. IQR: inter-quartile range. SD: standard deviation;

**Table 2:** Electrolytes profile and other laboratory findings of under-five diarrheal children with and without *Shigella* encephalopathy on admission.

Characteristics	Cases (n = 69) %	Controls (n = 70) %	Unadjusted OR	Unadjusted 95% CI	*p value*
Hyponatremia	61 (88)	46 (65)	4.8	1.8–12.9	0.00
Hypernatremia	2 (3)	2 (3)	1.0	0.1–7.4	1.00
Hypokalemia	19 (27)	27 (39)	0.60	0.3–1.2	0.22
Hyperkalemia	2 (3)	1 (1)	2.0	0.2–23.2	0.61
Hypochloremia	12 (17)	9 (13)	1.4	0.5–3.6	0.61
Metabolic acidosis	40 (58)	35 (50)	1.3	0.7–2.7	0.44
Hypoglycemia	7 (10)	0 (0)	-	-	0.04
Hypocalcemia	12/49 (24)	0/5 (0)	-	-	0.57
Raised S. creatinine	57/64 (89)	55/60 (92)	0.7	0.2–2.5	0.76
Anemia	34/68 (50)	44 (63)	0.5	0.3–1.1	0.18
Colitis in stool microscopy	37/63 (59)	39/61 (64)	0.8	0.4–1.6	0.68

**Table 3:** Stool isolates of *Shigella* children with & without encephalopathy.

Types of *Shigella*	Cases (n = 69) (%)	Controls (n = 70) (%)	Unadjusted OR	Unadjusted 95% CI	*p value*
*Shigella flexneri*	40 (58)	48 (69)	0.6	0.3–1.3	0.26
*Shigella sonnei*	24 (35)	10 (14)	3.2	1.4–7.4	0.00
*Shigella boydii*	5 (7)	7 (10)	0.7	0.2–2.3	0.76
*Shigella species*	0 (0)	5 (7)	0.0	-	0.05

In logistic regression analysis after adjusting for potential confounders, children with *Shigella* encephalopathy were independently associated with dehydrating diarrhea having shorter duration prior to admission, sepsis and hyponatremia (Table 4)

**Table 4:** Results of logistic regression analysis to explore independent risk factors for childhood *Shigella* encephalopathy.

Characteristics	Adjusted OR	Adjusted 95% CI	*p value*
Age (months)	0.9	0.9–1.0	0.078
Shorter duration of diarrhea prior admission	5.2	1.7–15.6	0.003
Dehydration (some/severe)	3.1	1.1–8.3	0.021
Severe acute malnutrition	0.4	0.1–1.1	0.088
Sepsis	7.5	2.3–24.3	0.001
Hyponatremia	3.5	1.0–12.3	0.048
*Shigella sonnei*	2.9	0.9–9.6	0.073

## Discussion

Shigellosis is one of the leading causes of mortality and morbidity in developing countries worldwide, with the highest geographical burden of disease in Asia [4,27]. Encephalopathy is one of the potentially fatal extra-intestinal complications, and here we describe clinical and laboratory characteristics of children less than five years of age associated with *Shigella* encephalopathy.

The most important observation of this study was the significantly higher deaths in children with *Shigella* encephalopathy compared to those without encephalopathy. Other important observations were the independent association of *Shigella* encephalopathy with dehydrating diarrhea having shorter duration prior to admission, sepsis, and hyponatremia.

Our observation of higher deaths in children with *Shigella* encephalopathy compared to those without encephalopathy is consistent with previous observations [5,10,11,28]. Although, overall case-fatality-rate among hospitalized shigellosis children reduced to <1% especially due to several non-specific interventions such as measles vaccination, vitamin A supplementation, and improved nutrition [29], the higher deaths despite the non-specific interventions in children having *Shigella* encephalopathy might be due to increased disease severity of such children. The mechanism of death from *Shigella* encephalopathy is yet to be well understood, even though it is often associated with cerebral edema or brain hemorrhage [8,13,18].

The association of shorter duration of diarrhea prior admission and dehydrating diarrhea has also been reported previously [10,11]. The shorter duration of diarrhea was probably due to early manifestation of encephalopathic features resulting in timely hospitalization [10]. Dehydration may be related to decreasing oral intake in an unconscious and or convulsing child.

Sepsis is a clinical syndrome that complicates severe infection. The overall burden of sepsis in children is high globally. The incidence of sepsis has been steadily rising since the mid-1990 with significant regional differences [30–32]. Our multivariate analysis showed that sepsis was independently associated with *Shigella* encephalopathy. Patient with acute shigellosis showed large local production of inflammatory cytokines such as TNF-alpha and IL-1 [21]. These pro-inflammatory cytokines may spill into the blood stream leading to more generalized inflammatory response [21,33]. Endothelial injury induced by reactive oxygen species, vasoactive substances such as nitric oxide, endothelin, platelet-derived growth factor (PDGF) may disrupt blood brain barrier in septic patients [34]. Dysfunction of the blood brain barrier probably contributes, allowing exposure to toxic mediators, increased leukocyte infiltration, and active transport of cytokines across the barrier. CNS dysfunction has also been attributed to changes in metabolism and alterations in cell signaling due to inflammatory mediators [35].

Hyponatremia is common in children with shigellosis and triggered by multiple factors [5,36]. Previously Khan et al showed association of higher serum potassium and lower serum sodium in children with shigellosis also having unconsciousness and convulsion, respectively [10]. Our observed association of hyponatremia with *Shigella* encephalopathy might be related to the inappropriate release of antidiuretic hormone (ADH). Children with *Shigella* encephalopathy were more volume-depleted as evident by the presence of dehydration which might stimulate baroreceptors leading to an increase in hormone secretion [36,37]. A child with *Shigella* encephalopathy is already at risk of having cerebral edema and raised intra cranial pressure [8,13,18]. The additional water movement into the brain from even a small fall in serum sodium can be lethal [38]. It has been demonstrated that in children with a variety of central nervous system diseases, hyponatremia is associated with neurological deterioration and poor outcome [39,40]. Prompt correction of hyponatremia in symptomatic patients has been considered critically important to reduce potential mortality and morbidity [41]. Hypertonic saline infusion establishes an osmotic gradient that favors water movement from the intracellular space into the intravascular space, reducing intracranial pressure. There is also evidence that hypertonic saline can improve cerebral edema by down-regulating glial cell water channels [42]. McNab et al demonstrated that use of isotonic fluid as maintenance intravenous fluid therapy lowered the risk of developing hyponatremia with little evidence of an increase in adverse events compared to those who received hypotonic fluid [43].

Observed association of hypoglycemia with *Shigella* encephalopathy is understandable and has been reported previously [10]. We observed children in encephalopathy group were relatively older, which is consistent with previous study [11]. Our uni-variate analysis showed children with *Shigella* encephalopathy were less likely to have SAM. In uni-variate analysis, we further observed that the infection with *Shigella sonnei* was significantly associated with encephalopathy, although many previous studies have reported no association between the infecting species of *Shigella* with encephalopathy [10,11,44]. Our observed association might represent the rising trend and increasing virulence of *Shigella sonnei* [1,45,46]. We also observed *Shigella sonnei* as the second most common species of *Shigella* among our study population [1], which might represent the improvement of overall nutritional and socioeconomic status. This relationship might explain our observed association of better nourished children in the encephalopathy group.

We observed a high percentage of *Shigella* strains were resistant to commonly used antibiotics such as trimethoprim-sulphamethoxazole, ciprofloxacin, and ampicillin [4]. In Bangladesh the resistance of *Shigella sonnei* to ciprofloxacin is increasing dramatically. It has been shown that in 2007, 10% of the strains were resistant to ciprofloxacin, and by 2011, the resistant strains peaked to 70% [1]. We also observed the similar trend i.e. more than nine folds (90–96%%) increased in resistant strains in our present data compared to those in 2007. This observation suggests appropriate measures should be taken to minimize antimicrobial resistance in low and middle-income countries including Bangladesh. Proper and judicious use of antibiotics should be practiced and simultaneously, over-the-counter sale of antibiotics without prescription should be prohibited that may reduce the emergence of antibiotic resistance in Asian countries [4]. Other important measures include restricted use of antibiotics in animal foods, education of allied health care professionals and public regarding unique features of bacterial infection and antibiotics, prudent antibiotic prescribing as a positive construct, and personal hygiene [47,48].

The main limitation is the retrospective nature of the study where we used registry data for analysis and it has limited a number of our variables of interest that might be associated with any of the study groups. Another limitation is the lack of performance of imaging studies that may help to document cerebral edema.

In conclusion, the results of our data suggest that the case fatality rate was significantly higher among children younger than five years of age with *Shigella* encephalopathy, compared to those without encephalopathy. Our results demonstrate that *Shigella* encephalopathy was independently associated with dehydrating diarrhea having shorter duration prior admission, sepsis, and hyponatremia. The results emphasize the importance of early identification of these simple features of *Shigella* encephalopathy and prompt use of appropriate antimicrobials as well as rehydration with sodium containing isotonic fluid may help clinicians to reduce morbidity & deaths in such children especially in resource limited settings.
